# Regulation of LH/FSH expression by secretoglobin 3A2 in the mouse pituitary gland

**DOI:** 10.1007/s00441-014-1794-z

**Published:** 2014-02-11

**Authors:** Yuki Miyano, Shigeyuki Tahara, Ichiro Sakata, Takafumi Sakai, Hiroyuki Abe, Shioko Kimura, Reiko Kurotani

**Affiliations:** 1 Biochemical Engineering, Faculty of Engineering, Yamagata University, Yonezawa, Yamagata 992-8510 Japan; 2 Department of Neurosurgery, Nippon Medical School, Tokyo, 113-8603 Japan; 3 Area of Regulatory Biology, Division of Life Science, Graduate School of Science and Engineering, Saitama University, Saitama, 338-8570 Japan; 4 Laboratory of Metabolism, National Cancer Institute, National Institutes of Health, Bethesda, MD 20892 USA

**Keywords:** SCGB3A2, Nkx2-1, C/EBP, LH/FSH, Pituitary

## Abstract

Secretoglobin (SCGB) 3A2 was originally identified as a downstream target for the homeodomain transcription factor NKX2-1 in the lung. NKX2-1 plays a role in the genesis and expression of genes in the thyroid, lung and ventral forebrain; *Nkx2*-*1*-null mice have no thyroid and pituitary and severely hypoplastic lungs and hypothalamus. To demonstrate whether SCGB3A2 plays any role in pituitary hormone production, NKX2-1 and SCGB3A2 expression in the mouse pituitary gland was examined by immunohistochemical analysis and RT-PCR. NKX2-1 was localized in the posterior pituitary lobe, whereas SCGB3A2 was observed in both anterior and posterior lobes as shown by immunohistochemistry and RT-PCR. Expression of CCAAT-enhancer binding proteins (C/EBPs), which regulate mouse *Scgb3a2* transcription, was also examined by RT-PCR. C/EBPβ, γ, δ and ζ were expressed in the adult mouse pituitary gland. SCGB3A2 was expressed in the anterior and posterior lobes from postnatal days 1 and 5, respectively and the areas where SCGB3A2 expression was found coincided with the area where FSH-secreting cells were found. Double-staining for SCGB3A2 and pituitary hormones revealed that SCGB3A2 was mainly localized in gonadotrophs in 49 % of FSH-secreting cells and 47 % of LH-secreting cells. In addition, SCGB3A2 dramatically inhibited LH and FSH mRNA expression in rat pituitary primary cell cultures. These results suggest that SCGB3A2 regulates FSH/LH production in the anterior pituitary lobe and that transcription factors other than NKX2-1 may regulate SCGB3A2 expression.

## Introduction

NKX2-1, also called TTF1, TITF1, or T/EBP, is a homeodomain-containing DNA-binding protein that was originally identified as a transcription factor regulating thyroid-specific expression of genes. NKX2-1 is expressed in the lung, thyroid and ventral forebrain during embryogenesis (Guazzi et al. 1990; Lazzaro et al. 1991; Mizuno et al. 1991) and plays a role in morphogenesis of these organs. Thus, NKX2-1 suppression by antisense oligonucleotides *in vitro* using embryonic lung organ cultures, inhibits branching morphogenesis (Minoo et al. 1995). Furthermore, targeted disruption of the *Nkx2*-*1* gene results in immediate postnatal death from respiratory failure caused by profoundly hypoplastic lungs (Kimura et al. 1996). In addition, *Nkx2*-*1*-null mice lack the thyroid and pituitary gland and exhibit severe defects in the ventral forebrain, including the hypothalamus and basal ganglia (Kimura et al. 1996; Takuma et al. 1998; Minoo et al. 1999; Sussel et al. 1999; Yuan et al. 2000). In *Nkx2*-*1*-null mice, a Rathke’s pouch rudiment initially forms during pituitary development but is eliminated by programmed cell death before formation of a definitive pouch (Takuma et al. 1998). In the diencephalon of the mutant, *Fgf8* expression, which is necessary for activation of a key regulatory gene, *Lhx3* and subsequent development of the pouch rudiment into a definitive pouch, is absent (Takuma et al. 1998). In the pituitary gland, NKX2-1 is expressed in the posterior lobe of fetal and adult rats, suggesting that NKX2-1 is directly associated with development of the posterior lobe of the pituitary gland (Nakamura et al. 2001).

Secretoglobin 3A2 (SCGB3A2), also called uteroglobin-related protein 1 (UGRP1), was originally identified as a downstream target for NKX2-1 in the lung through suppressive subtractive library screening of mRNAs isolated from lungs of *Nkx2*-*1*-null and wild-type mouse fetuses (Niimi et al. 2001). SCGB3A2 expression is mainly found in bronchial epithelial cells and is first detected in mouse fetal lungs at embryonic day (E) 11.5. Its expression markedly increases by E16.5 and continues in the airway epithelial cells (Clara cells) at relatively high levels throughout adulthood (Niimi et al. 2001; Kurotani et al. 2008; Tomita et al. 2008). Expression of SCGB3A2 is directly regulated by NKX2-1 together with C/EBPs as determined by luciferase reporter assays, gel shift and ChIP analyses in vitro (Tomita et al. 2008). In the lungs, SCGB3A2 plays multiple roles including promotion of both early and late stages of fetal lung development (Kurotani et al. 2008) and suppression of allergic airway inflammation (Chiba et al. 2006) and bleomycin-induced pulmonary fibrosis (Kurotani et al. 2011). However, the expression and role of SCGB3A2 in regions other than the lungs are not known. In this study, the expression of SCGB3A2 and its relationship to NKX2-1 expression is investigated in the mouse pituitary gland.

## Materials and methods

### Animals

C57BL/6 N mice (7–12 weeks old) were maintained under a 12-h light/dark cycle with free access to water and conventional food. Room temperature was maintained at 22 ± 1 °C. Embryos at embryonic day (E) 11.5, E13.5, E16.5 and E18.5 were obtained by cesarean section from pregnant mice and neonates at postnatal day (P) 1 and 5 (P5) were obtained after birth. All animal experiments were performed according to the Using Animals in Intramural Research Guidelines (Yamagata University School of Medicine) and approved by the Committee for Animal Experimentation.

### Immunohistochemistry

Mice were sacrificed under deep anesthesia with diethylether between 1000 and 1100 hours. The mouse pituitary glands and neonates were fixed by overnight immersion in 4 % paraformaldehyde in phosphate buffer (PB) at 4 °C. Samples were dehydrated with a series of ascending concentrations of ethanol, immersed in xylene and embedded in paraffin. Sections of 4 μm thickness were prepared, mounted on glass slides (Matsunami Glass, Osaka, Japan) and incubated in 0.3 % H_2_O_2_ in methanol for 30 min to inactivate endogenous peroxidases. Sections were then incubated in citrate buffer (pH 6.0) at 100 °C for 10 min, blocked in 5 % skim milk for 1 h at room temperature and incubated with primary antibody at 4 °C overnight in a humidified chamber. After washing three times for 5 min each in phosphate-buffered saline (PBS), sections were processed by the ABC method using a commercially available kit (Vector Laboratories, CA, USA) according to the manufacturer’s instructions. Immunocomplexes were visualized with 3,3′-diaminobenzidine tetrahydrochloride (DAB) (DAKO, Glostrup, Denmark). For double staining, the DAB-stained sections were blocked in 5 % skim milk for 1 h at room temperature followed by incubation with second primary antibody at 4 °C overnight. Sections were processed by the ABC method and immunocomplexes were visualized with Vector blue (Vector Laboratories). To determine the co-localization of SCGB3A2 and anterior pituitary hormones, 5 fields of ×400 magnifications of the frontal pituitary section (about 25 % of the anterior pituitary area) were randomly selected and SCGB3A2-immunopositive cells and double-immunopositive cells were counted. The percentage of double-immunopositive cells within SCGB3A2-immunopositive cells was then calculated and the data from 4–8 mice were averaged for each anterior hormone.

### Antibodies

Anti-NKX2-1 monoclonal antibody was obtained from Abcam (ab76013; Tokyo, Japan) that was produced in rabbits immunized with a synthetic peptide corresponding to residues near the N-terminus of human NKX2-1. Specificity was confirmed by immunoblotting and immunohistochemistry according to the manufacture’s data sheet. Anti-SCGB3A2 antibody was from R&D Systems (AF3465; Minneapolis, MN, USA), which was produced in goats immunized with a recombinant mouse SCGB3A2 peptide (amino acids 22–104). Anti-GH and PRL antibodies from Santa Cruz Biotechnology (GH: sc-10365, PRL: sc-7805; Santa Cruz, CA, USA), were produced in goats immunized with peptide mapping near the C-terminus of human GH and PRL, respectively. Anti-TSHβ, LHβ and FSHβ antisera were provided by Dr. T. Matozaki (Fujiwara et al. 2007). Anti-TSHβ antiserum was produced in rabbit immunized with purified rat TSHβ protein, which recognizes only β-subunit of TSH. Anti-LH and FSH antisera were produced in rabbits immunized with purified canine LH and FSH proteins respectively. Anti-ACTH serum obtained from Yanaihara Institute (Y352; Shizuoka, Japan) was produced in rabbits immunized with a synthetic mouse and rat ACTH peptide (amino acids 1–23).

### RT- PCR

Adult mice bone marrow, livers, lungs, pituitary glands, spleens, thymuses and embryonic lungs at embryonic day (E) 16.5 were removed and immersed in a TRIzol Reagent (Life Technologies, CA, USA). Pituitary glands were divided into anterior and intermediate-posterior lobes. Total RNA extracted was subjected to DNase treatment to eliminate genomic DNA and reverse-transcription (RT) using the RT Reagent kit (Takara Bio, Shiga, Japan) according to the manufacturer’s instructions. To detect *Scgb3a2*, PCR was performed using Amplitaq DNA Polymerase (Applied Biosystems, Branchburg, NJ, USA). Thermal condition used was 94 °C for 2 min followed by 40 or 45 cycles of 94 °C for 20 s, 60 °C for 30 s, 72 °C for 30 s and 1 cycle of 72 °C for 2 min. To detect C/EBPs, TaKaRa Ex Taq Hot Start Version (Takara Bio) was used with the thermal conditions of 94 °C for 5 min followed by 35 cycles of 94 °C for 20 s, 60 °C for 30 s, 72 °C for 30 s and 1 cycle of 72 °C for 2 min. The primers used for PCR are summarized in Table 1. 18S was used as a positive control in all tissues examined. The PCR products were electrophoresed on 1.5 % agarose gels and visualized by ethidium bromine staining under UV light.

**Table 1:** Primers for RT-PCR

Gene	Primer sequence	Product size (bp)
SCGB3A2	FWD: GACTGCATTCCAAAGTCCCG	111
	REV: GAGAAGGGCAGTGGCAGAATAACC	
18S	FWD: CGGCTACCACATCCAAGGAA	193
	REV: ATTGGAGCTGGAATTACCGC	
C/EBPα	FWD: CAAGAACAGCAACGAGTACCG	124
	REV: GTCACTGGTCAACTCCAGCAC	
C/EBPβ	FWD: GTTTCGGGACTTGATGCAAT	122
	REV: CCCGCAGGAACATCTTTAAGT	
C/EBPγ	FWD: GAGAATGAACGGTTGGAAGC	126
	REV: TGTAGTTTCCGTGCTGATGG	
C/EBPδ	FWD: ATCGACTTCAGCGCCTACAT	101
	REV: GCTTTGTGGTTGCTGTTGAA	
C/EBPε	FWD: CGCATTATGGAGACTCAGCA	102
	REV: GCGCAGAGTGTCTAGCTCCT	
C/EBPζ	FWD: AATCCAGGATGATGCTGTCC	140
	REV: TGTCTGGCAGAAGGTCTGTG	
LH	FWD: CTGAGCCCAAGTGTGGTGT	127
	REV: CACAGATGCTGGTGGTGAAG	
FSH	FWD: AAGTC ATCCAGCTTTGCAT	158
	REV: TCCCTGGTGTAGCAGTAGCC	
β-actin	FWD: TGGCACCACACTTTCTACAATGAG	106
	REV: GGGTCATCTTTTCACGGTTGG	

### Quantitative real-time RT-PCR

Total RNA from primary cells was prepared as described above. Messenger RNA expression was quantitatively measured using quantitative RT-PCR (qPCR) with Mx3000P Real-Time QPCR System (Agilent Technologies, Tokyo, Japan) and SYBR Premix Ex Taq II (Takara Bio). The primers used for qRT-PCR are summarized in Table 1. β-actin mRNA was used as a normalization control for LH and FSH mRNAs.

PCR was performed in triplicates with the thermal cycling conditions as follows: 95 °C for 10 min, followed by 40 cycles of 95 °C for 10 s, 60 °C for 30 s and 1 cycle of 95 °C for 1 min, 55 °C for 30 s and 95 °C for 30 s. For quantification, the comparative threshold cycle method was used to assess relative changes in mRNA levels between untreated (control) and SCGB3A2-treated samples (Livak and Schmittgen 2001).

### Primary culture and SCGB3A2 treatment of rat pituitary cells

Rats were anesthetized by an intraperitoneal injection of sodium pentobarbital (50 mg/kg) and perfused with Hank’s Balanced Salt Solution (HBSS) without calcium and magnesium (Life Technologies). Anterior pituitary glands were dissected and cut into pieces in HBSS containing 1 % trypsin (Life Technologies) and 0.2 % collagenase (Nitta Gelatin, Osaka, Japan). Tissue pieces were incubated at 37 °C in a water bath for 15 min. After centrifugation for 5 min at 2,000 rpm, the supernatant was removed and the tissue pieces were incubated in HBSS containing 1 % trypsin, 0.2 % collagenase and 5 μg/mL DNase I (QIAGEN, Tokyo, Japan) for 5 min at 37 °C. After centrifugation and removal of the supernatant, tissue pieces were incubated in HBSS containing 0.3 % EDTA (Wako Pure Chemical Industries, Osaka, Japan) for 5 min at 37 °C. Following centrifugation and removal of the supernatant, cells were dispersed in HBSS by pipetting and filtered through nylon mesh with 70 μm pores (BD, NJ, USA). Filtered cells were plated on 35-mm dishes (Greiner Bio-One, Frickenhausen, Germany) at a density of 5 × 10^5^ cells/dish in 2 ml of Medium 199 (Life Technologies) supplemented with 10 % fetal bovine serum (Life Technologies), 100 units/ml penicillin and 100 μg/ml streptomycin (Nacalai Tesque, Kyoto, Japan). Cells were cultured at 37 °C in a humidified atmosphere of 5 % CO_2_ and 95 % air. After 24 h of incubation, His-tagged recombinant mouse SCGB3A2 (300 ng/ml) was added to the media and plates were incubated for 24 h. Recombinant mouse SCGB3A2 was purified as described previously (Kurotani et al. 2008).

### Statistical analysis

Values are presented as means ± SE. Statistical analysis was performed using Student’s *t* test. *P* values of <0.05 were considered to be statistically significant.

## Results

### Localization of NKX2-1 in the adult mouse pituitary gland

**Fig. 1:**
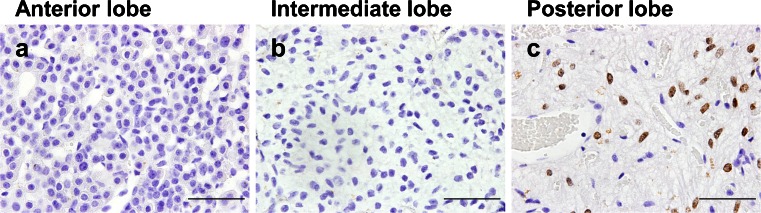
Expression of NKX2-1 in the adult mouse pituitary gland. Immunohistochemistry for NKX2-1 in the adult mouse pituitary gland (**a**–**c**). NKX2-1 was detected only in the nucleus of the posterior pituitary cells (**c**) but not in the anterior (**a**) or intermediate lobes (**b**). *Bars* 50 μm

Expression of NKX2-1 was examined by immunohistochemistry in the adult mouse pituitary gland. NKX2-1 expression was found only in the posterior lobes and not in the anterior or intermediate lobes of the pituitary gland as previously reported (Fig. 1) (Nakamura et al. 2001).

### Localization of SCGB3A2 in the mouse pituitary gland

**Fig. 2:**
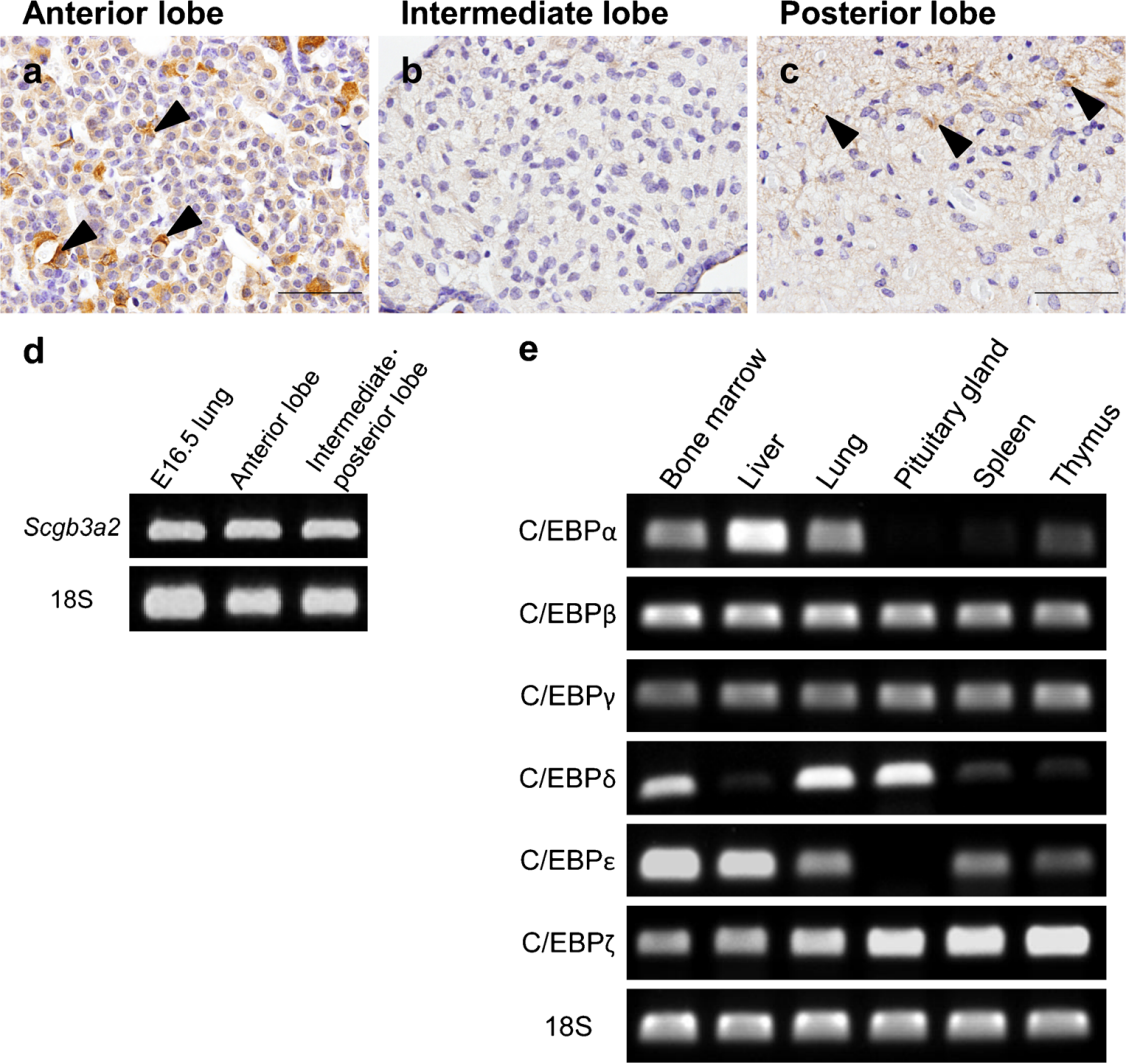
Expression of SCGB3A2 in the adult mouse pituitary gland. Immunohistochemistry for SCGB3A2 in the adult mouse pituitary gland (**a**–**c**). SCGB3A2 was detected in the anterior (**a**) and posterior lobes (**c**). *Bars* 50 μm. RT-PCR analysis of *Scgb3a2* mRNA in the adult mouse pituitary gland (**d**). cDNA samples were separately obtained from anterior and intermediate-posterior lobes. cDNA from mouse fetal lungs of E 16.5 was used as a positive control. *Scgb3a2* mRNA was detected in both anterior and intermediate-posterior lobes of the pituitary gland. RT-PCR analysis of mRNAs encoding C/EBPα-ζ was performed using cDNAs obtained from bone marrow, liver, lung, pituitary gland, spleen and thymus (**e**). In the pituitary gland, expression of C/EBPβ, γ, δ and ζ mRNAs were found, whereas no expression of mRNA encoding C/EBPα or ε was detected

Expression of SCGB3A2 in adult mouse pituitary gland was next examined by immunohistochemistry and RT-PCR. SCGB3A2 immunopositive cells were found in posterior as well as anterior lobes (Fig. 2a, c). *Scgb3a2* mRNA was detected by RT-PCR in both anterior and intermediate-posterior lobes (Fig. 2d). cDNAs obtained from mouse embryonic lungs at E16.5 were used as a positive control. These results demonstrated that SCGB3A2 is expressed in anterior and posterior lobes of pituitary gland. SCGB3A2 is directly regulated by NKX2-1 (Tomita et al. 2008). Taken together, these results suggest that transcription factors other than NKX2-1 may be involved in SCGB3A2 expression in the anterior pituitary. In a previous study, C/EBPs synergistically interacted with NKX2-1 to regulate mouse *Scgb3a2* transcription (Tomita et al. 2008). In order to determine whether C/EBPs are responsible for *Scgb3a2* expression, the expression of C/EBPs was examined by RT-PCR using cDNAs from the adult mouse pituitary gland. Because different tissues express different C/EBP isoforms (Ramji and Foka 2002), cDNAs obtained from bone marrow, liver, lung, spleen and thymus were used as controls. C/EBPβ, γ and ζ were detected at similar intensity levels in all tissues tested and conditions used (Fig. 2e). C/EBPδ was also expressed in all six tissues but the signals were stronger in bone marrow, lung and pituitary gland (Fig. 2e). C/EBPα and C/EBPε were not expressed in the pituitary gland (Fig. 2e).

### Expression of SCGB3A2 in the neonatal mouse pituitary gland

**Fig. 3:**
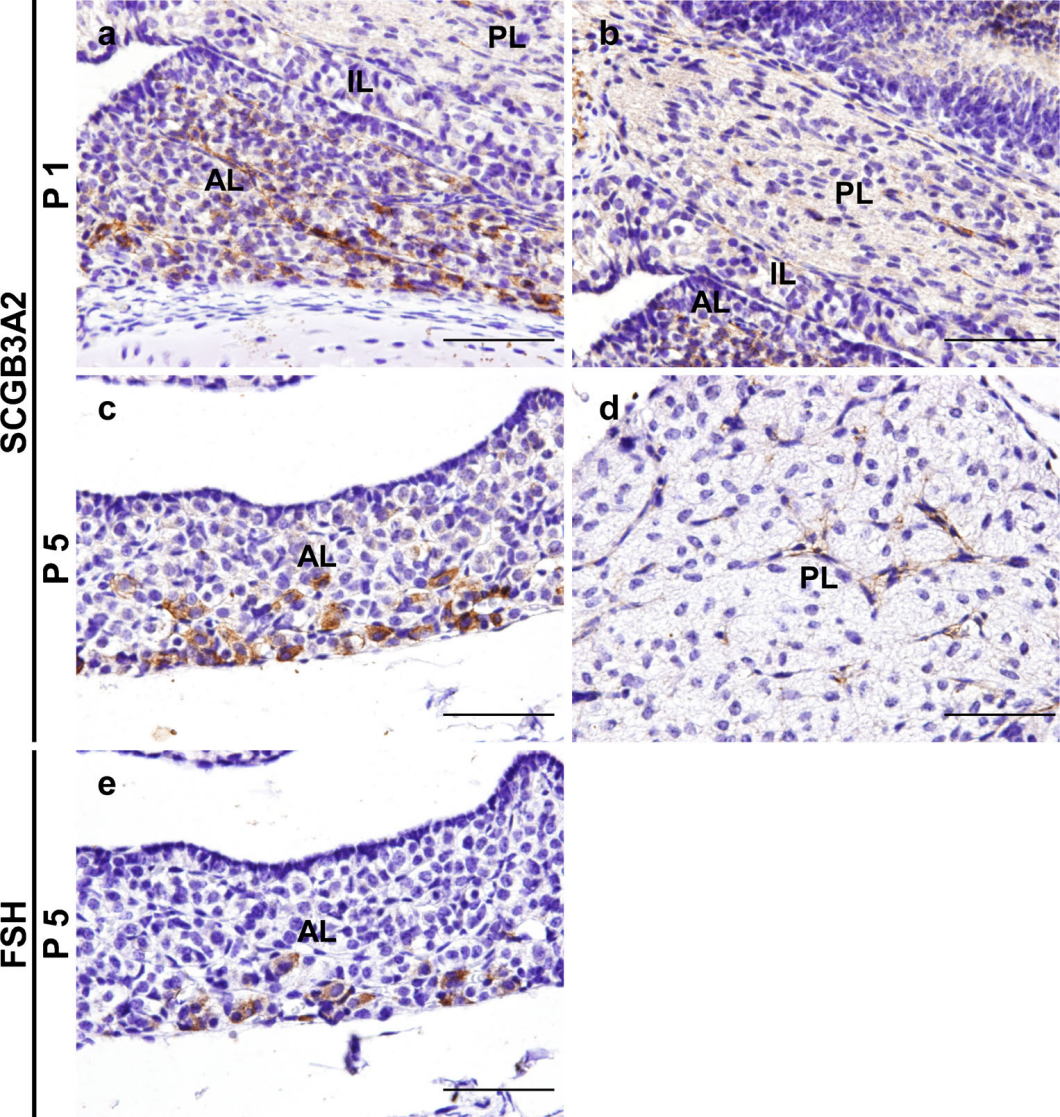
Expression of SCGB3A2 in the neonatal mouse pituitary gland. Immunohistochemistry for SCGB3A2 in postnatal day 1 (P1) (**a**, **b**) and postnatal day 5 (P5) (**c**, **d**) mouse pituitary glands. SCGB3A2 was detected in the anterior and posterior lobes from P1. Immunohistochemistry for FSH in P5 mouse pituitary glands using serial sections (**e**). FSH was detected in similar regions where SCGB3A2-immunopositive cells were present. *AL* anterior lobe, *IL* intermediate lobe, *PL* posterior lobe. *Bars* 50 μm

Although SCGB3A2 expression in fetal mouse lungs becomes detectable at E11.5 and markedly increases by E16.5 (Niimi, Keck-Waggoner et al. 2001), no clear signals were detected in the pituitary gland at E11.5, E13.5, E16.5 and E18.5 by immunohistochemistry (data not shown). Therefore, sagittal sections of neonatal mice at P1 and P5 were stained with SCGB3A2 antibody. Immunopositive cells were identified in anterior and posterior lobes of the pituitary gland at both P1 (Fig. 3a. b) and P5 (Fig. 3c. d); strong positive reactions were found in the ventral area of the anterior pituitary gland, particularly at P5. It was reported that expression of LHβ and FSHβ is found in the anteroventral area of the anterior pituitary at E16.5 and E17.5, respectively and the expressing cells extended posteriorly and laterally up to P1 (Japon et al. 1994). Therefore, FSH was stained using serial sections of P5 neonatal mice (Fig. 3e). The FSH-producing cells were localized in regions where SCGB3A2-immunopositive cells were present (Fig. 3c, e).

### Co-localization of SCGB3A2 and pituitary hormones

**Fig. 4:**
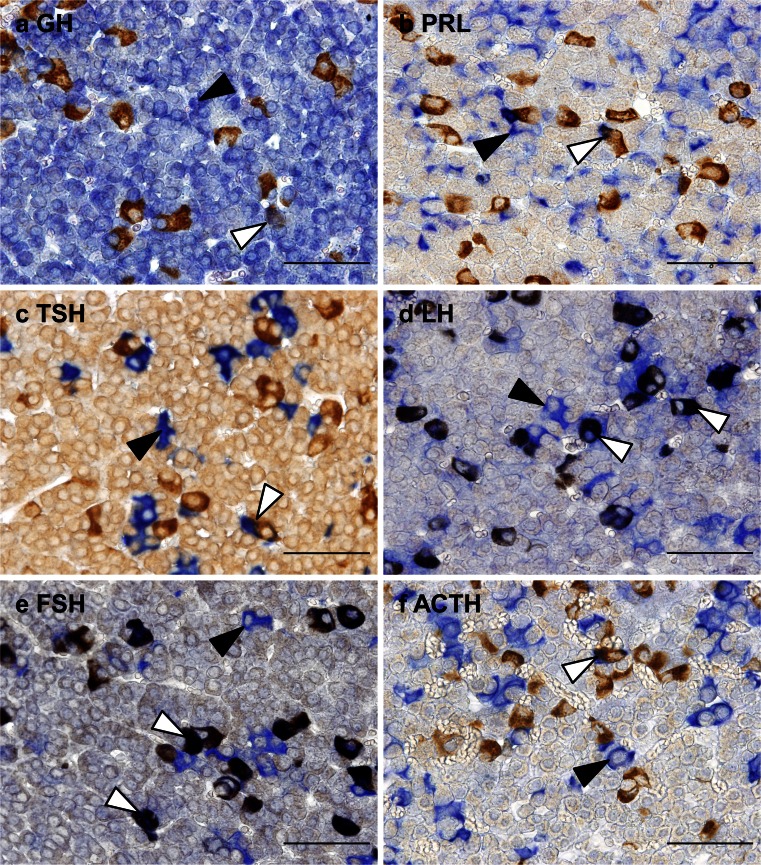
Double staining of SCGB3A2 and anterior pituitary hormones in the adult mouse pituitary gland. Immunohistochemistry for SCGB3A2 and growth hormone (**a**), prolactin (**b**), thyroid-stimulating hormone (**c**), luteinizing hormone (**d**), follicle-stimulating hormone (**e**) and adrenocorticotropic hormone (**f**). *Brown signal* indicates SCGB3A2, whereas *blue signal* indicates anterior pituitary hormones. *Black arrowheads* indicate immunopositive cells for anterior pituitary hormones and *white arrowheads* indicate double-positive cells for SCGB3A2 and anterior pituitary hormones. *Bars* 50 μm

To identify the type of SCGB3A2-expressing cells in the anterior lobe of the adult mouse pituitary gland, double staining for SCGB3A2 and pituitary hormones was performed. SCGB3A2 was well co-localized with gonadotrophs expressing LH and FSH and double-immunopositive cells were found at 47.3 % ± 9.9 and 48.7 % ± 7.7 %, respectively (Fig. 4; Table 2). GH-, PRL-, TSH- and ACTH-immunopositivety was found in 5.1 % ± 1.4, 1.5 % ± 0.2, 5.8 % ± 0.6 and 14.4 % ± 1.3 % of SCGB3A2-immunopositive cells, respectively (Fig. 4; Table 2).

**Table 2:** Immunohistochemical co-localization of SCGB3A2 and pituitary hormones

	Hormone					
	GH	PRL	TSH	LH	FSH	ACTH
SCGB3A2-expressing cells (%)	5.1 ± 1.4	1.5 ± 0.2	5.8 ± 0.6	47.3 ± 9.9	48.7 ± 7.7	14.4 ± 1.3

The adult mouse pituitary glands were immunostained with SCGB3A2 and each anterior pituitary hormones-specific antibody and immunopositive cells of 5 fields of ×400 magnification were counted. Nearly half of the SCGB3A2 immunostained cells were LH- and FSH-positive and to some extent ACTH-positive

Few SCGB3A2 immunostained cells were positive for GH, PRL and TSH. GH, LH, FSH, ACTH, *n* = 4; TSH, *n* = 5; PRL, *n* = 8

### Effects of SCGB3A2 stimulation on mRNA expression of gonadotrophs

**Fig. 5:**
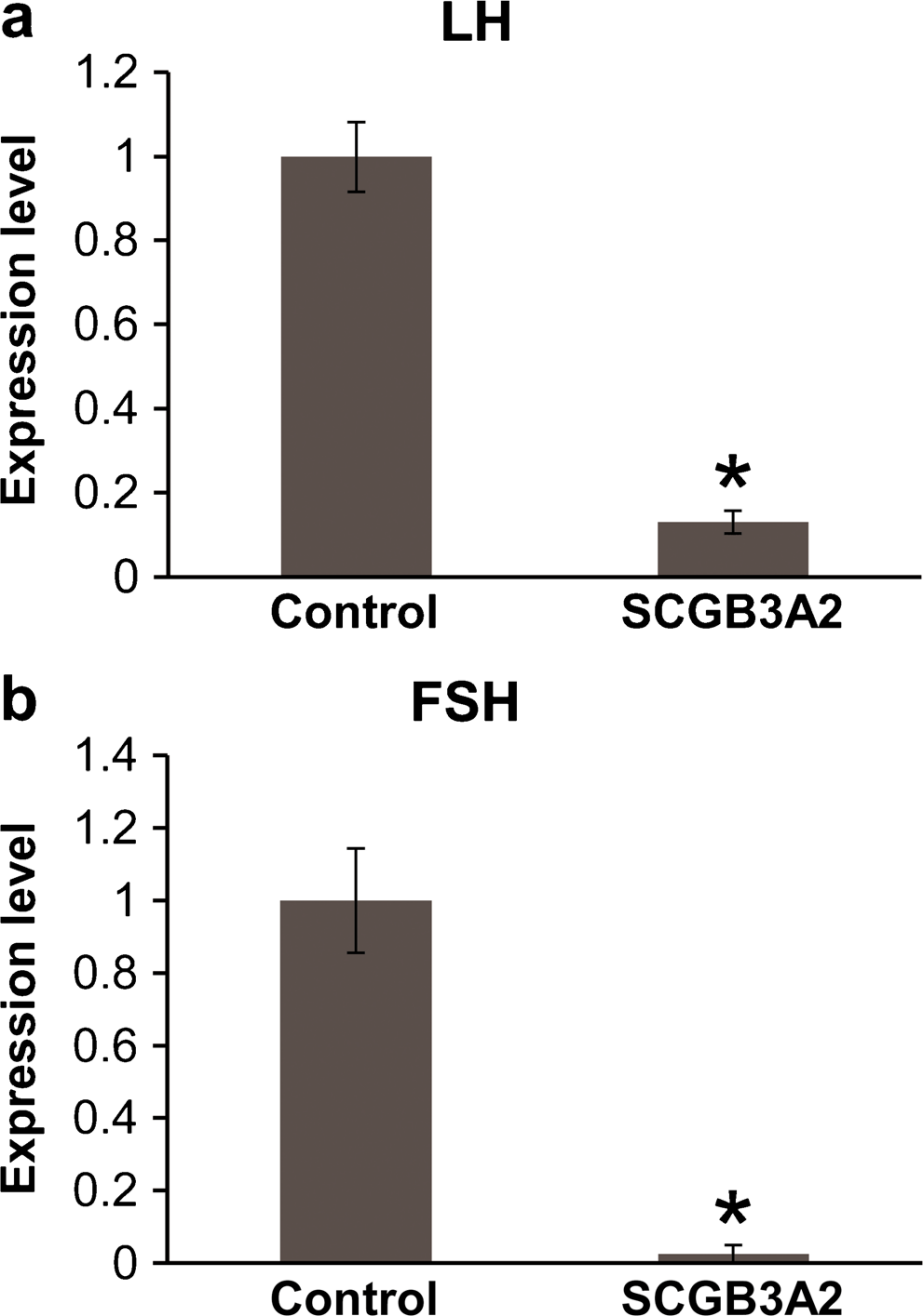
Effects of SCGB3A2 stimulation on mRNA expression of anterior pituitary hormones in primary cultured rat pituitary cells. Rat anterior pituitary cells were cultured and stimulated by SCGB3A2 (300 ng/ml) for 24 h and mRNA were extracted. Expression of LH (**a**) and FSH (**b**) was quantified by real-time RT-PCR. Expression levels of LH and FSH were significantly inhibited by SCGB3A2 stimulation. *P < 0.01; *n* = 6–8

To clarify the role of SCGB3A2 expressed in the anterior pituitary gland, the effect of SCGB3A2 on mRNA expression of LH and FSH in primary cultured cells of the rat anterior pituitary was investigated using qPCR. Stimulation of SCGB3A2 (300 ng/ml) significantly inhibited LH and FSH expression (*P* < 0.01) (Fig. 5).

## Discussion

This is the first report showing the presence of SCGB3A2 in the mouse pituitary gland. In this study, immunohistochemistry and RT-PCR revealed that SCGB3A2 is expressed in both the anterior and posterior lobes of the pituitary gland, whereas NKX2-1 is expressed only in the posterior lobe, as previously reported (Nakamura et al. 2001). Considering that SCGB3A2 is a direct target for NKX2-1 (Niimi et al. 2001; Tomita et al. 2008), these results suggest that SCGB3A2 expression is regulated by transcription factors other than NKX2-1 in the anterior lobe of the pituitary gland. Indeed, it was reported that C/EBPα and C/EBPδ regulate *Scgb3a2* transcription in mice by binding to specific sites located in the *Scgb3a2* promote and the activity is synergistically enhanced through cooperative interaction with NKX2-1 (Tomita et al. 2008). C/EBPs are a family of transcription factors containing the basic leucine zipper (bZIP) domain at the C-terminus that is involved in dimerization and DNA binding (Ramji and Foka 2002). Six members of the family, C/EBPα, β, γ, δ, ε and ζ, have been identified to date and they play pivotal roles in controlling cellular proliferation and differentiation, metabolism, inflammation and numerous other responses, particularly in hepatocytes, adipocytes and hematopoietic cells (Ramji and Foka 2002). Few studies have investigated the potential roles of C/EBPs in pituitary cell lines. For example, C/EBPα was detected in extracts of GH-secreting GC cells and prolactin-secreting 235-1 cells (Lew et al. 1993). In addition, expression of exogenous C/EBPα in GHFT1-5 cells activated a co-transfected GH gene promoter (Schaufele et al. 2001) and prolonged the cell cycle (Liu et al. 2002). Furthermore, C/EBPα and Pit-1 cooperated in the activation of both PRL and GH transcription (Schaufele et al. 2001; Enwright et al. 2003). Finally, C/EBPβ and C/EBPδ were shown to activate the clusterin promoter and induced clusterin protein expression was evident in gonadotroph cells and pituitary tissue overexpressing pituitary tumor transforming gene (PTTG) (Chesnokova et al. 2011). These reports indicate that C/EBPs are important for control of expression of pituitary hormones; however, to the best of our knowledge, C/EBP expression in the mouse pituitary gland has not been previously reported. In this study, C/EBPβ, γ, δ and ζ were clearly expressed in the adult mouse pituitary gland. Taken together, these studies suggest that C/EBPs and/or C/EBP-regulated pituitary-specific transcription factors may regulate *Scgb3a2* transcription in the pituitary gland, particularly in the anterior lobe of the pituitary gland.

SCGB3A2 expressed in the anterior pituitary gland co-localized with gonadotrophs in adult and neonatal mice and SCGB3A2 suppressed the expression of LH and FSH mRNAs in primary cultures of rat pituitary cells. These results suggest that SCGB3A2 may regulate FSH/LH production in an autocrine or paracrine manner. SCGB3A2 is a secreted protein and exhibits growth factor, anti-inflammatory and anti-fibrotic activities (Chiba et al. 2006; Kurotani et al. 2008; Kurotani et al. 2011). However, the mechanisms of these activities, including the involvement of a possible SCGB3A2 receptor, have not been elucidated. Macrophage scavenger receptor with collagenous structure (MARCO) expressed in alveolar macrophages in the lungs has been suggested to be a receptor for SCGB3A2 (Bin et al. 2003); however, one report identified a possible SCGB3A2-specific receptor on the mesenchymal cells of mouse fetal lungs where MARCO could not be found (Kurotani et al. 2008). Therefore, further studies are required to understand the mechanisms of action of SCGB3A2 including identification of its receptor(s) and the downstream associated signaling pathways.

The finding that both NKX2-1 and SCGB3A2 are expressed in the posterior lobe of the pituitary suggests that NKX2-1 regulates *Scgb3a2* transcription in the posterior pituitary and thus may have a role in posterior pituitary function. In rats, NKX2-1 is expressed in the pituicyte of the posterior lobe (Nakamura et al. 2001). Although pituicytes are the astrocyte-type glial cells that have long been suspected of having a role in regulating the secretion of neurohypophysial hormones (Leveque and Small 1959), the exact function of the pituicyte and the role of NKX2-1 in the pituicyte remain unknown. It would be interesting to investigate the role of NKX2-1 and/or SCGB3A2 in the regulation of neurohypophysial hormone release.

In conclusion, SCGB3A2 may regulate FSH/LH production in the anterior lobe of the pituitary gland and transcription factors other than NKX2-1 likely control expression of *Scgb3a2* in this organ.
